# Supplementary material for: WikiGenomes: an open web application for community consumption and curation of gene annotation data in Wikidata
Source: Database (Oxford). 2017 Mar 24;2017:bax025. doi: 10.1093/database/bax025 (PMC5467579; doi:10.1093/database/bax025)
Supplement: Supplementary Data [file bax025_Supp.docx]

**Supplemental File 1 SPARQL queries used in WikiGenomes.org**

**Endpoint:** https://query.wikidata.org/sparql

**# Get all bacterial strains from Wikidata with a genome and genes**

PREFIX wdt: <http://www.wikidata.org/prop/direct/>

PREFIX wd: <http://www.wikidata.org/entity/>

SELECT ?species ?speciesLabel ?taxid ?RefSeq

WHERE {

?species wdt:P171* wd:Q10876; # P171: Parent taxon

wdt:P685 ?taxid; # P685: NCBI Taxonomy ID

wdt:P2249 ?RefSeq. # P2249: NCBI RefSeq Genome ID

SERVICE wikibase:label {

bd:serviceParam wikibase:language "en" .

}

}

**# Get all genes and proteins (identifiers and genomic annotations) for a given taxid**

PREFIX pq: <http://www.wikidata.org/prop/qualifier/>

PREFIX p: <http://www.wikidata.org/prop/>

PREFIX wdt: <http://www.wikidata.org/prop/direct/>

PREFIX wd: <http://www.wikidata.org/entity/>

SELECT ?gene ?specieswd ?specieswdLabel ?taxid ?genomeaccession ?geneLabel

?locustag ?entrezid ?genomicstart ?genomicend ?strand ?protein ?proteinLabel

?uniprot ?refseqProtein ?refSeqChromosome

WHERE {

?specieswd wdt:P685 "115713";

wdt:P685 ?taxid; # P685: NCBI Taxonomy ID

wdt:P2249 ?genomeaccession. # P2249: NCBI RefSeq Genome ID

?gene wdt:P703 ?specieswd; # P703: Found in taxon

wdt:P351 ?entrezid ; # P351: Entrez Gene ID

wdt:P644 ?genomicstart; # P644: Genomic Start Position

wdt:P645 ?genomicend; # P645: Genomic End Position

wdt:P2393 ?locustag; # P2393: NCBI Locus Tag

wdt:P2548 ?strand; # P2548: Genomic Strand Orientation

p:P644 ?chr.

OPTIONAL{

?gene wdt:P688 ?protein. # P688: Encodes

?chr pq:P2249 ?refSeqChromosome. # P2249: NCBI RefSeq Genome ID

?protein wdt:P352 ?uniprot; # P352: UniProt ID

wdt:P637 ?refseqProtein. # P637: RefSeq Protein ID

}

SERVICE wikibase:label {

bd:serviceParam wikibase:language "en" .

}

}

**# Get the operon and other genes under its regulation for a given entrez gene id**

PREFIX wdt: <http://www.wikidata.org/prop/direct/>

SELECT ?gene ?locusTag ?entrez ?operon ?operonLabel ?genStart ?genEnd ?strand

?strandLabel ?op_genes ?op_genesLabel

WHERE {

?gene wdt:P351 '895005'; # P351: Entrez Gene ID

wdt:P361 ?operon. # P361: Part of

?operon wdt:P527 ?op_genes; # P527: has part

wdt:P2393 ?locusTag; # P2393: NCBI Locus Tag

wdt:P351 ?entrez; # P351: Entrez Gene ID

wdt:P644 ?genStart; # P644: Genomic Start Position

wdt:P645 ?genEnd; # P645: Genomic End Position

wdt:P2548 ?strand. # P2548: Genomic Strand Orientation

SERVICE wikibase:label {

bd:serviceParam wikibase:language "en" .

}

}

**# Get all go evidence code items in Wikidata**

PREFIX skos: <http://www.w3.org/2004/02/skos/core#>

SELECT DISTINCT ?evidence_code ?evidence_codeLabel ?alias ?eviURL

WHERE {

?evidence_code wdt:P31 wd:Q23173209; # P31: instance of

skos:altLabel ?alias;

wdt:P856 ?eviURL. # P856: Official Website

filter (lang(?alias) = "en")

SERVICE wikibase:label {

bd:serviceParam wikibase:language "en" .

}

}

**# Get all GO terms, the determination method for the annotation, the reference and**

**# enzyme class number for a given uniprot ID**

PREFIX ps: <http://www.wikidata.org/prop/statement/>

PREFIX prv: <http://www.wikidata.org/prop/reference/value/>

PREFIX pq: <http://www.wikidata.org/prop/qualifier/>

PREFIX p: <http://www.wikidata.org/prop/>

PREFIX wdt: <http://www.wikidata.org/prop/direct/>

PREFIX wd: <http://www.wikidata.org/entity/>

SELECT ?protein ?proteinLabel ?goterm ?reference_stated_inLabel ?reference_retrievedLabel

?determination ?determinationLabel ?gotermValue ?gotermValueLabel ?goclass ?goclassLabel

?goID ?ecnumber ?pmid

WHERE {

?protein wdt:P352 "Q9Z8Y5". # P352: UniProt ID

{?protein p:P680 ?goterm} # P680: Molecular Function

UNION {?protein p:P681 ?goterm} # P681: Biological Process

UNION {?protein p:P682 ?goterm}. # P682: Cellular Component

?goterm pq:P459 ?determination;

prov:wasDerivedFrom/pr:P248 ?reference_stated_in; # P248: Stated in

prov:wasDerivedFrom/pr:P813 ?reference_retrieved . # P813: retrieved

OPTIONAL {

?goterm prov:wasDerivedFrom/pr:P698 ?pmid . # P698: PMID

}

{?goterm ps:P680 ?gotermValue}

UNION {?goterm ps:P681 ?gotermValue}

UNION {?goterm ps:P682 ?gotermValue}.

?gotermValue wdt:P279* ?goclass; # P279: Subclass of

wdt:P686 ?goID.

FILTER ( ?goclass = wd:Q2996394 || ?goclass = wd:Q5058355 || ?goclass = wd:Q14860489)

OPTIONAL {

?gotermValue wdt:P591 ?ecnumber. # P591: EC Number

}

SERVICE wikibase:label {

bd:serviceParam wikibase:language "en" .

}

}

**# Return GO Terms with search term “kinase” in the label**

SELECT DISTINCT ?goterm ?goID ?goterm_label

WHERE {

?goterm wdt:P279* wd:Q14860489;

rdfs:label ?goterm_label;

wdt:P686 ?goID. # P686: Gene Ontology ID

FILTER(lang(?goterm_label) = "en")

FILTER(CONTAINS(LCASE(?goterm_label), ‘kinase’))

}

**# Get all InterPro domains and their references for a given uniprot id**

PREFIX prv: <http://www.wikidata.org/prop/reference/value/>

PREFIX ps: <http://www.wikidata.org/prop/statement/>

PREFIX p: <http://www.wikidata.org/prop/>

PREFIX wdt: <http://www.wikidata.org/prop/direct/>

PREFIX wd: <http://www.wikidata.org/entity/>

SELECT DISTINCT ?protein ?interPro_item ?interPro_label ?ipID

?reference_stated_inLabel ?pubDate ?version ?refURL

WHERE {

?protein wdt:P352 "Q9Z8Y5";

p:P527 ?interPro. # P527: has part

?interPro ps:P527 ?interPro_item;

prov:wasDerivedFrom/pr:P248 ?reference_stated_in; # P248: Stated in

prov:wasDerivedFrom/pr:P577 ?pubDate; # P577: Publication Date

prov:wasDerivedFrom/pr:P348 ?version; # P348: Software Version

prov:wasDerivedFrom/pr:P854 ?refURL . # P854: Reference URL

?interPro_item wdt:P2926 ?ipID;

rdfs:label ?interPro_label.

SERVICE wikibase:label {

bd:serviceParam wikibase:language "en" .

}

filter (

lang(?interPro_label) = "en") .

}
